# CTNNAL1 promotes the structural integrity of bronchial epithelial cells through the RhoA/ROCK1 pathway: CTNNAL1 promotes the structural integrity of BECs

**DOI:** 10.3724/abbs.2024026

**Published:** 2024-04-11

**Authors:** Caixia Liu, Jinmei Wang, Yurong Tan, Chi Liu, Xiangping Qu, Huijun Liu, Meiling Tan, Changqing Deng, Xiaoqun Qin, Yang Xiang

**Affiliations:** 1 Key Laboratory of Hunan Province for Integrated Traditional Chinese and Western Medicine on Prevention and Treatment of Cardio-Cerebral Diseases Hunan University of Chinese Medicine Changsha 410208 China; 2 School of Basic Medicine Central South University Changsha 410078 China

**Keywords:** CTNNAL1, human bronchial epithelial cells, structural integrity, ozone, RhoA/ROCK1

## Abstract

Adhesion molecules play critical roles in maintaining the structural integrity of the airway epithelium in airways under stress. Previously, we reported that catenin alpha-like 1 (CTNNAL1) is downregulated in an asthma animal model and upregulated at the edge of human bronchial epithelial cells (HBECs) after ozone stress. In this work, we explore the potential role of CTNNAL1 in the structural adhesion of HBECs and its possible mechanism. We construct a CTNNAL1 ^‒/‒^ mouse model with CTNNAL1-RNAi recombinant adeno-associated virus (AAV) in the lung and a *CTNNAL1*-silencing cell line stably transfected with CTNNAL1-siRNA recombinant plasmids. Hematoxylin and eosin (HE) staining reveals that CTNNAL1 ^‒/‒^ mice have denuded epithelial cells and structural damage to the airway. Silencing of *CTNNAL1* in HBECs inhibits cell proliferation and weakens extracellular matrix adhesion and intercellular adhesion, possibly through the action of the cytoskeleton. We also find that the expressions of the structural adhesion-related molecules E-cadherin, integrin β1, and integrin β4 are significantly decreased in ozone-treated cells than in vector control cells. In addition, our results show that the expression levels of RhoA/ROCK1 are decreased after *CTNNAL1* silencing. Treatment with Y27632, a ROCK inhibitor, abolished the expressions of adhesion molecules induced by ozone in CTNNAL1-overexpressing HBECs. Overall, the findings of the present study suggest that CTNNAL1 plays a critical role in maintaining the structural integrity of the airway epithelium under ozone challenge, and is associated with epithelial cytoskeleton dynamics and the expressions of adhesion-related molecules via the RhoA/ROCK1 pathway.

## Introduction

Adhesion molecules on the airway epithelium are a large family of transmembrane receptors that have been identified to be important for maintaining epithelial integrity, maintaining homeostasis, and promoting the inflammatory response [ 1– 3]. Destruction of bronchial epithelial integrity induced by abnormal adhesion is critical for asthma pathogenesis [ 4, 5]. In our previous study, we found that the expression of catenin alpha-like 1 (CTNNAL1) is downregulated in asthma patients and in an ovalbumin-stressed asthmatic mouse model, but markedly increased in human bronchial epithelial cells (HBECs) in the lungs and at the edge of HBECs exposed to ozone stress [ 6– 8]. Moreover, CTNNAL1 has been shown to promote wound repair in bronchial epithelial cells (BECs), inhibit ozone-induced airway epithelial-mesenchymal transition, and regulate mucus hypersecretion induced by house dust mite (HDM) [ 9, 10]. These data indicate that CTNNAL1 may have a protective effect on airway epithelial homeostasis.

The maintenance of respiratory microenvironment homeostasis depends on the airway epithelium. In the presence of environmental pollutants and allergens, the airway epithelium not only acts as an initial barrier but also participates in inflammatory activation. An increasing number of studies have shown that airway epithelial cells play a central role in the pathogenesis of airway diseases, such as asthma and chronic obstructive pulmonary disease (COPD) [ 11, 12]. The denudation of ciliated cells in asthma patients suggests that the airway epithelial barrier is often compromised [ 12, 13]. Therefore, we hypothesized that CTNNAL1 may be involved in the structural integrity of airway epithelial cells.

To verify this hypothesis, we constructed a mouse model with CTNNAL1-RNAi recombinant adeno-associated virus (AAV) in lung tissue and stably transfected cell lines ( *CTNNAL1* knockdown and overexpression) and investigated the effects of CTNNAL1 on the proliferation, cell-matrix distribution and cell-cell adhesion of HBECs. We detected the expressions of the structural adhesion-related molecules E-cadherin, integrin β1, and integrin β4. We further assessed the role of CTNNAL1, which is a component of the Rho GTPase complex [13], in the actin cytoskeleton and the RhoA/Rho-associated coiled-coil-containing protein kinase (ROCK1) signaling pathway in HBECs. We propose that CTNNAL1 contributes to epithelial integrity by regulating RhoA/ROCK1 signaling and plays a protective role in the functional adhesion of HBECs.

## Materials and Methods

### Mouse models establishment ozone stress

Male C57BL/6 mice, 4 weeks of age and free of murine‐specific pathogens, were obtained from Hunan SJA Laboratory Animal Co., Ltd (Changsha, China) and housed under barrier conditions in air‐filtered, temperature‐controlled units with free access to food and water. All animal studies were approved by the Ethics Committee Institute of Clinical Pharmacology of the Central South University (No. 201803246). All the methods were carried out in accordance with the relevant guidelines and regulations of the Guide for the Care and Use of Laboratory Animals of the National Institutes of Health. Mice were intratracheally administered with AAV5 and AAV5-CTNNAL1 siRNA (5×10 ^9^  vg per mouse; GeneChem, Shanghai, China). Then, the mice were stressed with ozone (2.0 ppm, 30 min) for 4 days by using a commercial ozonator (Model LT-100; Litian, Beijing, China) and sacrificed 24 h after the last exposure, whereas the control mice (administered with AAV5) were treated with fresh air.

### Cell culture, stable transfection and model of ozone stress

The immortalized HBEC cell line 16HBE14o- was a kind gift from Dr Dieter C Gruenert (University of California, San Francisco, USA) [14]. The cells were maintained in a mixture of Dulbecco’s modified Eagle’s medium (DMEM):F12 (1:1) (Gibco, Carlsbad, USA) supplemented with 10% fetal bovine serum (Gibco), 100 U/mL penicillin and 100 μg/mL streptomycin and were incubated at 37°C in a humidified 5% CO _2_ atmosphere. An inhibitor (#S1049) used in subsequent experiments was purchased from Selleck Chemicals (Houston, USA).

As previously described [15], pcDNA3.1(–)/CTNNAL1, pGCU6/Neo/RFP/CTNNAL1-RNAi, pcDNA3.1(‒) or pGCU6/Neo/RFP (GeneChem, Shanghai, China) was transfected into 16HBE14o- cells using FuGENE1 HD transfection reagent (Roche Applied Science, Mannheim, Germany) according to the manufacturer’s protocol. Next, the transfected cells were incubated in medium containing 600 μg/mL of G418. After 14 days, the positive resistant clones were maintained in complete culture medium supplemented with 200 μg/mL of G418.

Stably transfected cells were passaged using 0.05% trypsin (Thermo Fisher Scientific, Waltham, USA), and seeded into coated six-well plates at a density of 1×10 ^5^ to 2×10 ^5^ cells per well. At approximately 50%‒60% confluence, the cells were exposed to 1.5 ppm ozone for 60 min.

### Cell viability assay

The effect of CTNNAL1 on cell viability was measured by the Cell Counting Kit (CCK-8) assay, as previously described [16]. Briefly, cells were counted, adjusted, and seeded in a 96-well plate with 6 replicates for each group. The stably transfected cells were treated with an equal volume (100 μL) of DMEM. According to the manufacturer’s protocol, CCK-8 (10 μL; Solarbio, Beijing, China **)** was added to each well and the cell culture plate was incubated for 1‒4 h at 37°C. Then, the absorbance at 450 nm was measured with a microplate reader (Thermo Fisher Scientific).

### Quantitative real-time PCR

Total RNA was extracted using RNAiso Plus (TaKaRa, Kusatsu, Japan) following the manufacturer’s protocol. Reverse transcription was performed using a RevertAid ^TM^ First Strand cDNA Synthesis Kit (Thermo Fisher Scientific). Subsequently, quantitative real-time PCR (qRT-PCR) was performed on an Applied Biosystems 1900 System (Applied Biosystems, Foster City, USA) using the SYBR Green I Real Time PCR Kit (Bio-Rad, Hercules, USA). Primers were synthesized according to the sequences listed in Table 1. The expression of the housekeeping gene ( *GAPDH*) was used to normalize the expressions of the target genes with approximately equal amplification efficiency.

**Table:** **Table 1** Sequences of the primers used in the qRT-PCR analysis

Gene	Primer sequence
*GAPDH*	Forward: 5′-GAAGGTGAAGGTCGGAGTC-3′
	Reverse: 5′-GAAGATGGTGATGGGATTTC-3′
*CTNNAL1*	Forward: 5′-GGAGTTTGCACATCTGAGTGGA-3′
	Reverse: 5′-CCAATGCCACTTTCATACGG-3′
*E-cadherin*	Forward: 5′-TCCAGGAACCTCTGTGATGGA-3′
	Reverse: 5′-ACTCTCTCGGTCCAGCCCA-3′
*Integrin β1*	Forward: 5′-CCGCGCGGAAAAGATGAATTT-3′
	Reverse: 5′-CCACAATTTGGCCCTGCTTG-3′
*Integrin β4*	Forward: 5′-CACCTCCGTCTCCTCCCAC-3′
	Reverse: 5′-GTTGGGGATGTTGAGCCGA-3′
*RhoA*	Forward: 5′-AGTCCACGGTCTGGTCTTC-3′
	Reverse: 5′-TTCCACAGGCTCCATCAC-3′
*ROCK1*	Forward: 5′-AGGCATAAATCCACCAGGAA-3′
	Reverse: 5′-GCCATGATGTCCCTTTCTTC-3′

### Fluorescence-activated cell sorting analysis

The cells were harvested and treated with 1% Triton X-100 (Sigma-Aldrich, St Louis, USA), followed by washing and treatment with a mouse monoclonal antibody against CTNNAL1 (ab57875; Abcam, Cambridge, UK). Then, the cells were incubated with a phycoerythrin (PE)-labelled secondary antibody and analyzed by flow cytometry (FCM) on a MoFLo™ XDP cytometer (Beckman Coulter, Brea, USA).

### Cell cycle assay

After being cultured in 6-well plates, the cells were harvested, fixed, and stored at –20°C overnight. The fixed cells were washed, incubated with RNase A (Sigma-Aldrich), stained with propidium iodide (PI; Sigma-Aldrich), and analyzed by FCM. 16HBE14o- cells (PI-free) were used as a negative control.

### EdU incorporation assay

A Cell-Light TM EdU DNA Cell Proliferation kit (RiBoBio, Shanghai, China) was used to measure DNA synthesis. The cells were serum-starved and incubated with 50 μM of EdU. Then, the cells were harvested and fixed with 4% polyformaldehyde. The cells were stained with Apollo fluorescent dye (RiBoBio), permeabilized, and washed. Finally, the cells were resuspended in phosphate buffer solution (PBS) to determine the mean fluorescence intensity (MFI) of the EdU solution by FCM.

### Cell-matrix adhesion assay

The adherence of the extracellular matrix (ECM) to 16HBE14o- cells was assessed using 96-well culture plates coated with rat tail collagen. The cells were seeded and incubated at 37°C in 5% CO _2_ for 120 min. The adherent cells were incubated with MTT solution (Solarbio) **,** then the culture medium was removed, and dimethyl sulfoxide (Sigma-Aldrich) was added. The optical density (OD) was subsequently measured with a microplate reader (Varioskan Flash; Thermo Fisher Scientific). Five replicates of each sample were analyzed in each assay.

### Western blot analysis

Whole-cell lysates were prepared from cells using RIPA lysis buffer containing protease inhibitor cocktail (Thermo Fisher Scientific). Lysates were fractionated by 8%–10% SDS-PAGE and then transferred onto a polyvinylidene fluoride (PVDF) membrane (Merck Millipore, Darmstadt, Germany). After being blocked with 5% bovine serum albumin, the membranes were incubated with antibodies against GAPDH (60004-I-Ig; Proteintech, Rosemont, USA), CTNNAL1 (ab57875; Abcam), integrin β1 (BF0336; Affinity), integrin β4 (ab29042; Abcam), E-cadherin (3195s; Cell Signaling Technology, Danvers, USA), RhoA (ab187027; Abcam), or ROCK1 (ab45171; Abcam), followed by incubation with HRP-conjugated goat anti-mouse IgG (ab136815; Abcam) or goat anti-rabbit IgG (ab136817; Abcam) secondary antibody. The bands were detected using an enhanced chemiluminescence detection system (Merck Millipore) and a ChemiDoc XRS imaging system (Bio-Rad). GAPDH was used as a loading control.

### Cytoskeletal staining

After being fixed in 4% paraformaldehyde and permeated with 0.5% Triton X-100, the adherent cells were incubated with phalloidin-TRITC (diluted at 1:200; Yeasen Biotechnology, Shanghai, China) to label the F-actin filaments. Subsequently, the nuclei were stained with 4′,6-diamidino-2-phenylindole (DAPI; Sigma-Aldrich) for 2 min. The cytoskeleton was observed at 630× magnification using a laser scanning confocal microscope (LSW710; Zeiss, Oberkochen, Germany).

### Scanning electron microscopy analysis

Transfected cells were seeded on climb slices (Solarbio), fixed using fixation buffer (2% glutaraldehyde+4% paraformaldehyde) and dehydrated in a graded series of ethanol (70%‒100%). Subsequently, the specimens were vacuum-dried and sprayed with gold particles with an IB-5 Ion Coater (Eiko, Tokyo, Japan). The cell adhesion morphology was observed using a scanning electron microscope (S-3400 N; Hitachi, Tokyo, Japan).

### RhoA activity assay

A Rho activation assay kit (17-294; Merck Millipore) was used to measure RhoA activity. Briefly, cells were harvested and lysed in Mg ^2+^ lysis buffer. Then, the cell extract was incubated with the Rhotekin RBD agarose beads. The condensate in the tube bottom was subsequently collected for western blot analysis.

### Haematoxylin-eosin (HE) staining

The lung tissues of the mice were obtained and fixed with 4% paraformaldehyde. Then, the samples were dehydrated, transparent, wax dipped, embedded, and sliced. Subsequently, the sections were cut into 4-μm pathological sections and stained with haematoxylin and eosin (Beyotime Biotechnology, Shanghai, China) after dewaxing and rehydration. Pathological changes in the lung tissues were examined via a microscope (TS100; Nikon, Tokyo, Japan).

### Statistical analysis

All experiments were performed at least three times. The data were analyzed using SPSS 22.0 (SPSS Inc., Chicago, USA) and presented as the mean±standard error of the mean (SEM). The results were analyzed using one-way analysis of variance for multiple comparisons or a *t*-test for two groups. The differences were considered statistically significant when *P*<0.05.

## Results

### CTNNAL1 deficiency caused damage to the structural integrity of the airway

**Figure 1:**
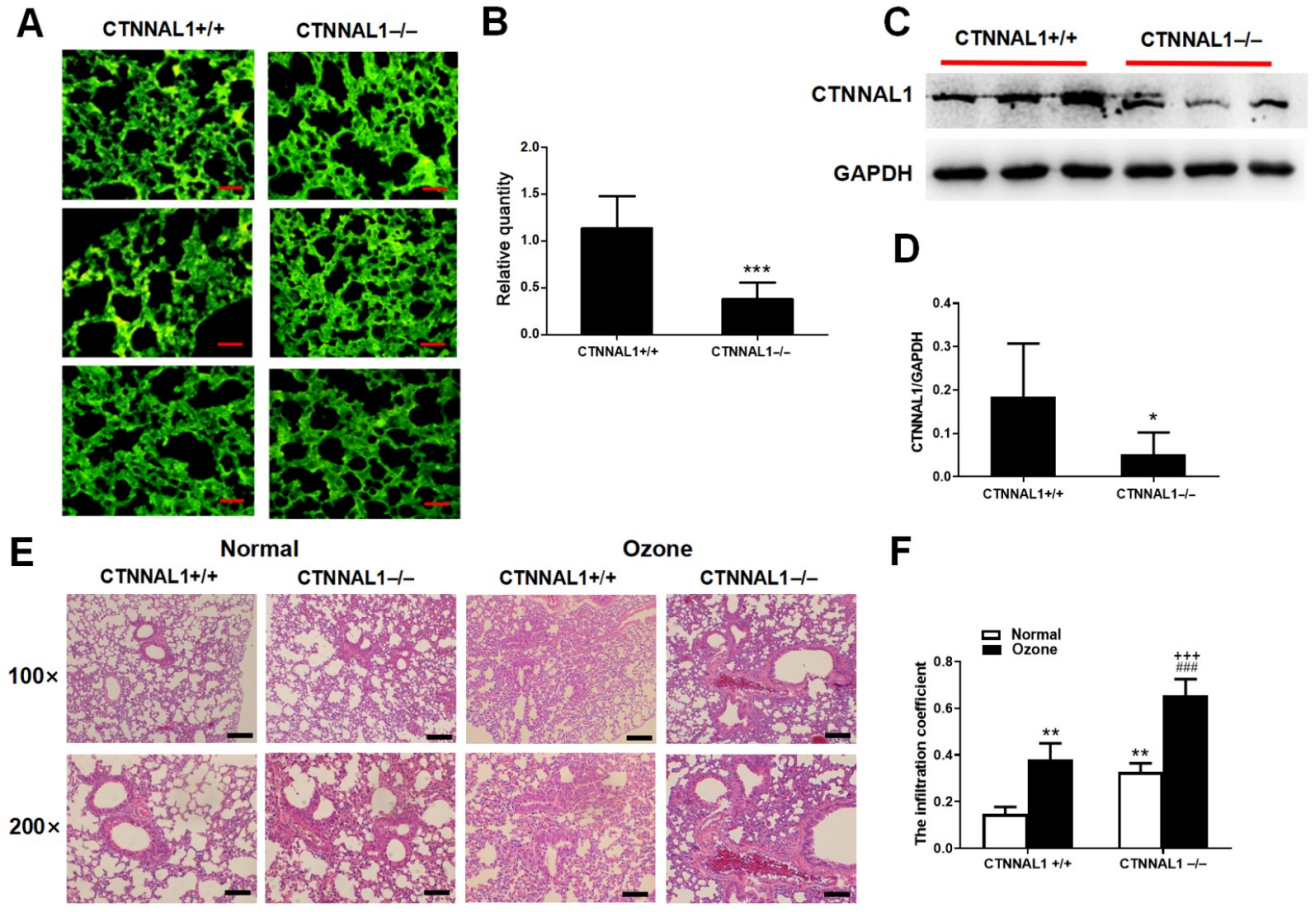
Figure 1 CTNNAL1 deficiency impaired the structural integrity of the airway *in vivo* First, CTNNAL1-knockdown mice were generated by intratracheal injection of recombinant AAV5-CTNNAL1-RNAi, and AAV5 was used as a vector control. (A) The infection efficiency of AAV5 and AAV5-CTNNAL1-RNAi in lung tissue was evaluated via the cryosectioning technique. Scale bar: 50 μm. (B) CTNNAL1 mRNA expression was detected via qRT-PCR. (C,D) The protein expression of CTNNAL1 was assessed by western blot analysis. (E) The structural integrity of the airway was subsequently determined via HE staining. Scale bars: 100 μm (100×), 50 μm (200×). (F) Analysis of the infiltration coefficient of lung tissue. *P<0.05, **P<0.01, ***P<0.001 vs CTNNAL1+/+; ###P<0.001 vs CTNNAL1‒/‒; +++P<0.001 vs CTNNAL1+/++ozone. n=6.

To determine the role of CTNNAL1 in the structural integrity of the airway, we constructed a mouse model with recombinant AAV5-CTNNAL1-RNAi in bronchopulmonary tissue. First, we observed the infection efficiency of AAV5 and AAV5-CTNNAL1-RNAi using cryosections of lung tissue. The fluorescence intensity in the lungs of the mice in each group indicated successful and efficient infection with AAV ( Figure 1A). Moreover, the mRNA and protein expressions of CTNNAL1 in CTNNAL1 ^–/–^ mice were significantly reduced ( Figure 1B‒D). HE staining revealed that CTNNAL1 ^–/–^ mice had denudation of epithelial cells, structural damage to the airway and a greater infiltration coefficient of lung tissue ( Figure 1E,F). Notably, the damage to airway structural integrity and inflammation in lung tissue of CTNNAL1 ^–/–^ mice were markedly aggravated compared to those of CTNNAL1 ^+/+^ mice after ozone exposure, indicating that the damage to airway integrity and lung inflammation induced by ozone was increased in CTNNAL1 ^–/–^ mice.

### The structural adhesion of 16HBE14o- cells was weakened after CTNNAL1 silencing

**Figure 2:**
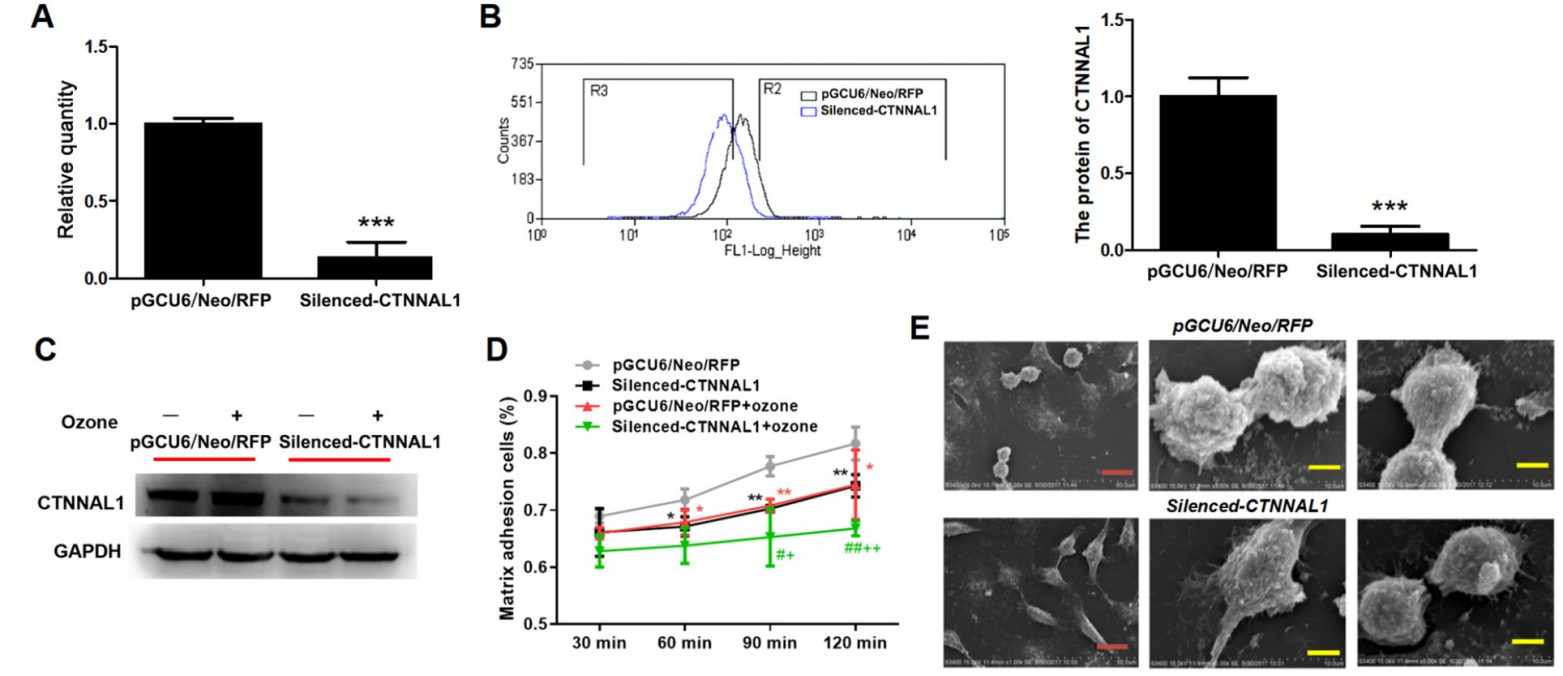
Figure 2 The structural adhesion of 16HBE14o- cells was decreased after *CTNNAL1* silencing *in vitro* A stable CTNNAL1-silenced HBEC cell line was constructed first and the cell-matrix adhesion and cell-cell adhesion were evaluated. (A,B) mRNA and protein expressions of CTNNAL1 were evaluated by qRT-PCR and FCM. (C) The protein level of CTNNAL1 in ozone-induced 16HBE14o- cells. (D) Percentage of epithelial cells adhering to rat tail collagen from 30 to 120 min with or without ozone exposure. (E) Scanning electron microscopy images of cell-cell adhesion ability. Data are presented as the mean±SEM of 3 independent experiments. *P<0.05, **P<0.01, ***P<0.001 vs pGCU6/Neo/RFP; #P<0.05, ##P<0.01 vs silenced-CTNNAL1, +P<0.05; ++P<0.01 vs pGCU6/Neo/RFP+ozone. n=3.

To further determine whether the expression of CTNNAL1 was essential for the structural integrity of airway epithelial cells, a stable *CTNNAL1*-silenced HBEC cell line was constructed with CTNNAL1-siRNA recombinant plasmids. qRT-PCR results showed that the CTNNAL1 mRNA expression was significantly lower in the *CTNNAL1*-silenced 16HBE14o- cells than in the vector control cells ( Figure 2A). The protein level of CTNNAL1 in the *CTNNAL1*-silenced cell line was reduced by more than 80%, as determined by FCM ( Figure 2B). We subsequently evaluated the effects of CTNNAL1 on matrix adhesion between 16HBE14o- cells and rat tail collagen from 30 min to 120 min. Its expression level did not increase in the *CTNNAL1*-silenced cells after exposure to ozone ( Figure 2C). Our results showed that *CTNNAL1* silencing caused a consistent decrease in the percentage of cells adherent to rat tail collagen, especially at 90 min and 120 min, while the adhesion of 16HBE14o- cells to rat tail collagen in the two groups was significantly inhibited by ozone ( Figure 2D). In addition, the intercellular adhesion ability of the cells was investigated by scanning electron microscopy. As shown in Figure 2E, after silencing of *CTNNAL1*, the intercellular junctions were impaired or even disrupted, indicating that the intercellular adhesion capacity was inhibited by *CTNNAL1* knockdown.

### CTNNAL1 deficiency decreased the levels of structural adhesion molecules in 16HBE14o- cells

**Figure 3:**
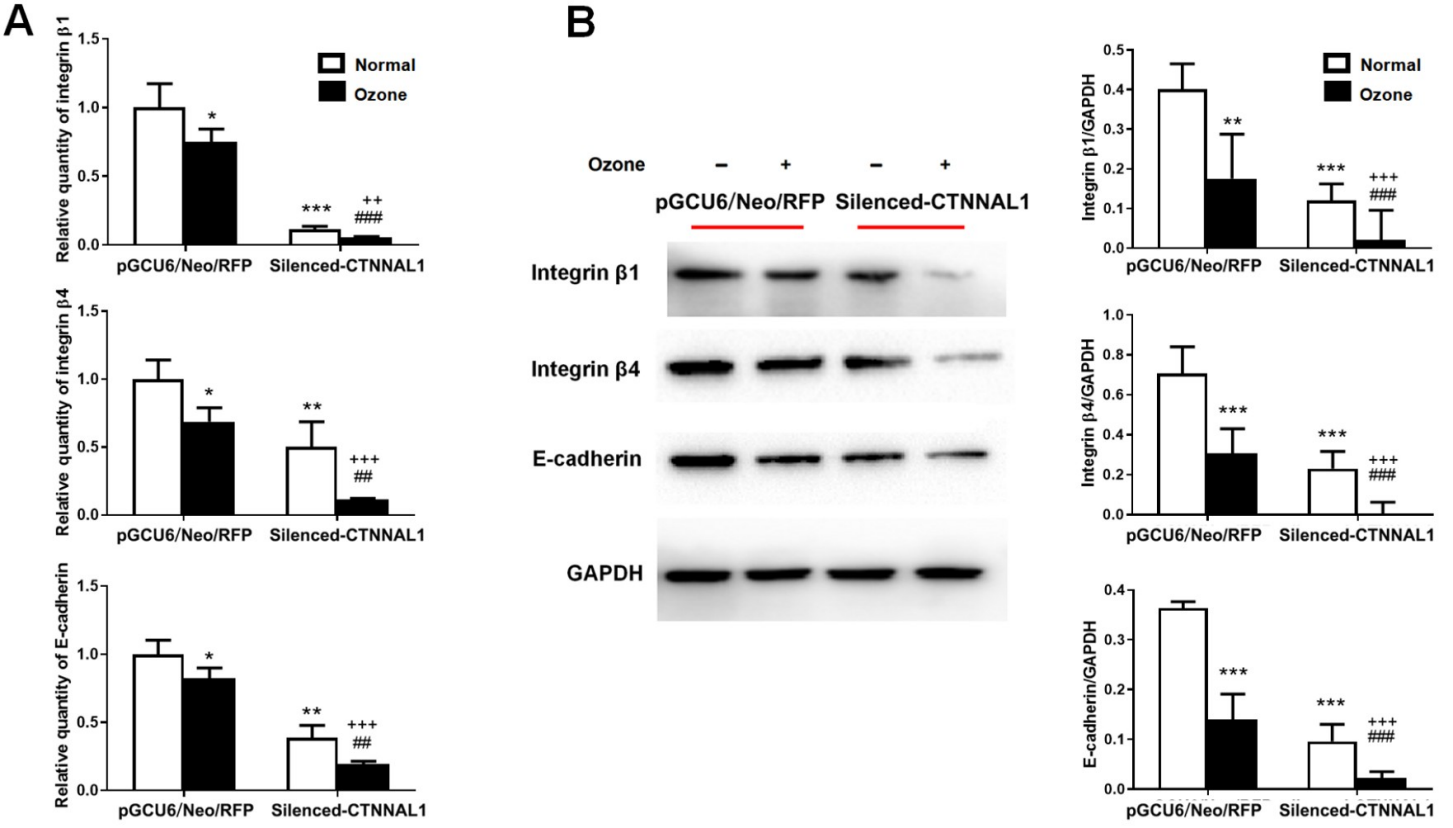
Figure 3 Expressions of adhesion molecules (A) The mRNA levels of structural adhesion molecules (integrin β1, integrin β4 and E-cadherin) were determined by qRT-PCR. n=5. (B) Western blot analysis of the protein expressions of integrin β1, integrin β4 and E-cadherin. n=3. *P<0.05, **P<0.01, ***P<0.001 vs pGCU6/Neo/RFP; ##P<0.01, ###P<0.001 vs silenced-CTNNAL1; ++P<0.01, +++P<0.001 vs pGCU6/Neo/RFP+ozone.

To further determine whether the decrease in CTNNAL1 expression is involved in the structural adhesion of airway epithelial cells under ozone stress, we examined the mRNA and protein expressions of the cell-cell adhesion molecule E-cadherin and the cell-matrix adhesion molecules integrin β1 and integrin β4 by qRT-PCR and western blot analysis, respectively. After *CTNNAL1* silencing, the mRNA expressions of E-cadherin, integrin β1, and integrin β4 were decreased by 88.53%, 48.66%, and 61.05%, respectively ( Figure 3A), while the protein expression shown in Figure 3B was consistent with the mRNA expression. Interestingly, the mRNA and protein expression levels of E-cadherin, integrin β1, and integrin β4 in *CTNNAL1*-silenced cells were significantly lower than those in pGCU6/Neo/RFP cells after ozone exposure, indicating that the ozone-induced decrease in the expressions of structural adhesion molecules was greater in the *CTNNAL1*-silenced cells ( Figure 3).

### CTNNAL1 promoted the proliferation of 16HBE14o- cells

**Figure 4:**
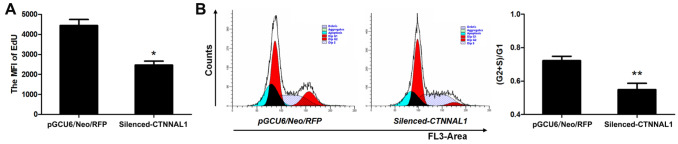
Figure 4 Effect of *CTNNAL1* silencing on the proliferation of 16HBE14o- cells (A) The MFI of EdU in 16HBE14o- cells was analyzed via FCM. n=3. (B) FACS plots and the percentage of 16HBE14o- cells in the (G2+S)/G1 phase. n=4. Data are expressed as the mean±SEM of at least one of the independent experiments performed in triplicate and were normalized to the vector control. *P<0.05, **P<0.01 vs pGCU6/Neo/RFP.

To explore the effects of CTNNAL1 on the proliferation of 16HBE14o- cells, the cells were first incubated with EdU. EdU can be incorporated into the DNA of proliferating mammalian cells [17], and its MFI is consistent with the proliferation ability of cells. The MFI of EdU in *CTNNAL1*-silenced cells was 44.56% lower ( Figure 4A) than that in pGCU6/Neo/RFP cells. These results indicated that CTNNAL1 may promote the proliferation of 16HBE14o- cells.

To further confirm the growth-promoting effect of CTNNAL1 on 16HBE14o- cells, we investigated changes in the cell cycle. The distribution of 16HBE14o- cells in different phases of the cycle was detected by measuring the intracellular DNA content. As shown in Figure 4B, the ratio of (G2+S)/G1 was decreased by 38.64% in the *CTNNAL1*-silenced cells compared with that in the pGCU6/Neo/RFP cells.

### CTNNAL1 affected the skeletal distribution

**Figure 5:**
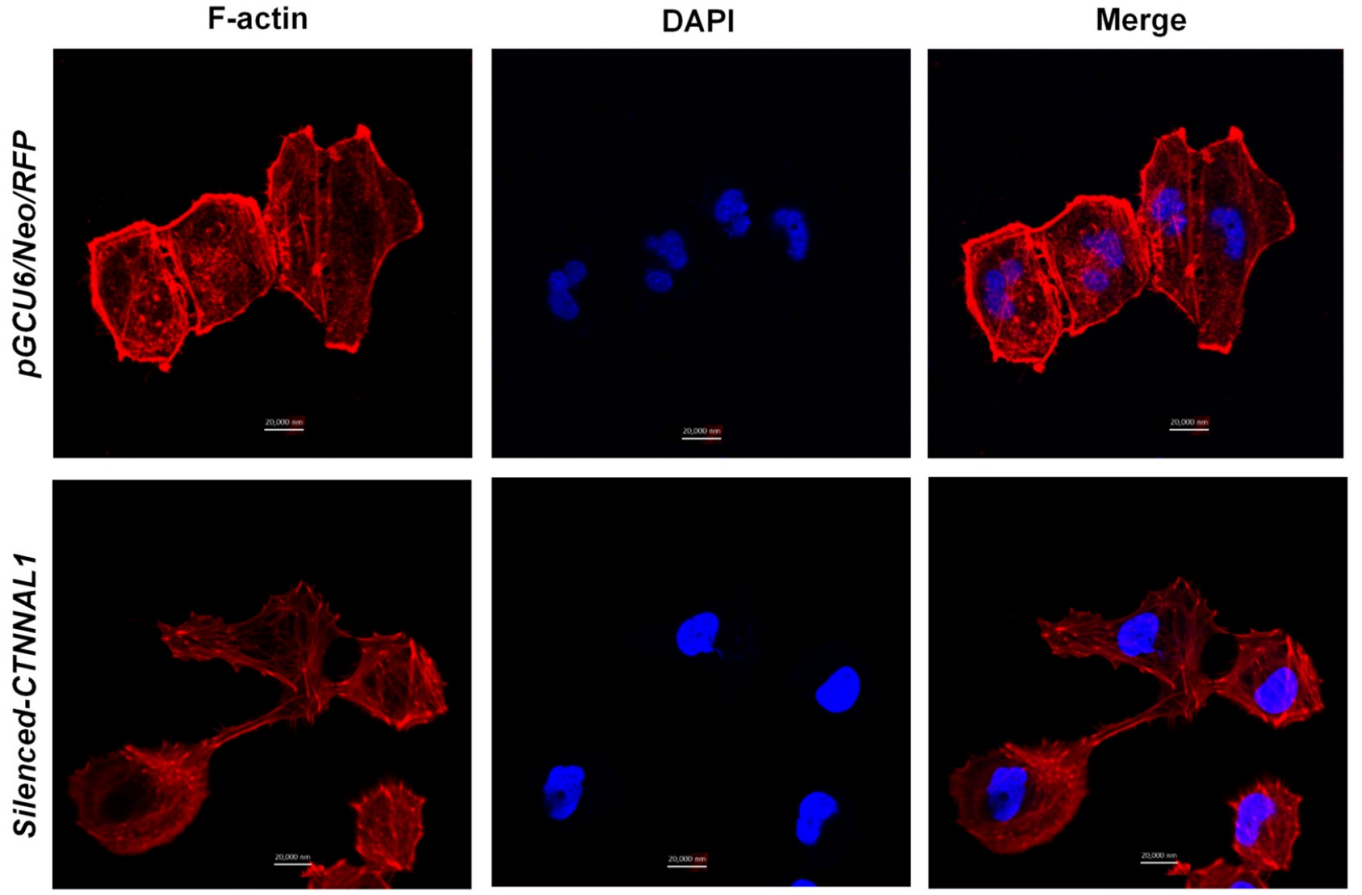
Figure 5 Imaging of the F-actin cytoskeleton (red) in vector control and *CTNNAL1*-silenced cells F-actin filaments were labelled with phalloidin-TRITC and the nuclei were stained with DAPI. The cytoskeleton was observed at 630× magnification using the laser scanning confocal microscope. n=3.

The adhesion of epithelial cells is inseparable from dynamic changes in the cytoskeleton. Therefore, after observing changes in cell-cell and cell-matrix adhesion, as well as in the expressions of adhesion-related molecules, we next assessed the effect of *CTNNAL1* silencing on the cytoskeletal arrangement of 16HBE14o- cells. Figure 5 shows the distribution of F-actin, a cytoskeletal filament involved in various important cell functions, such as the formation of cell pseudo feet, cell adhesion, and cell division. The results indicated that F-actin was located mainly around the cell periphery in the pGCU6/Neo/RFP group, while the continuity of peripheral F-actin fibers in the *CTNNAL1*-silenced group was significantly reduced and disrupted. Additionally, cells subjected to *CTNNAL1* silencing exhibited some filopodia and punctiform structures of fibers in their cell bodies. Moreover, we observed weakened intercellular junctions in the *CTNNAL1*-silenced group, which was consistent with the scanning electron microscopy images.

### CTNNAL1 promoted the expressions of RhoA and ROCK1 and elevated the activity of RhoA

**Figure 6:**
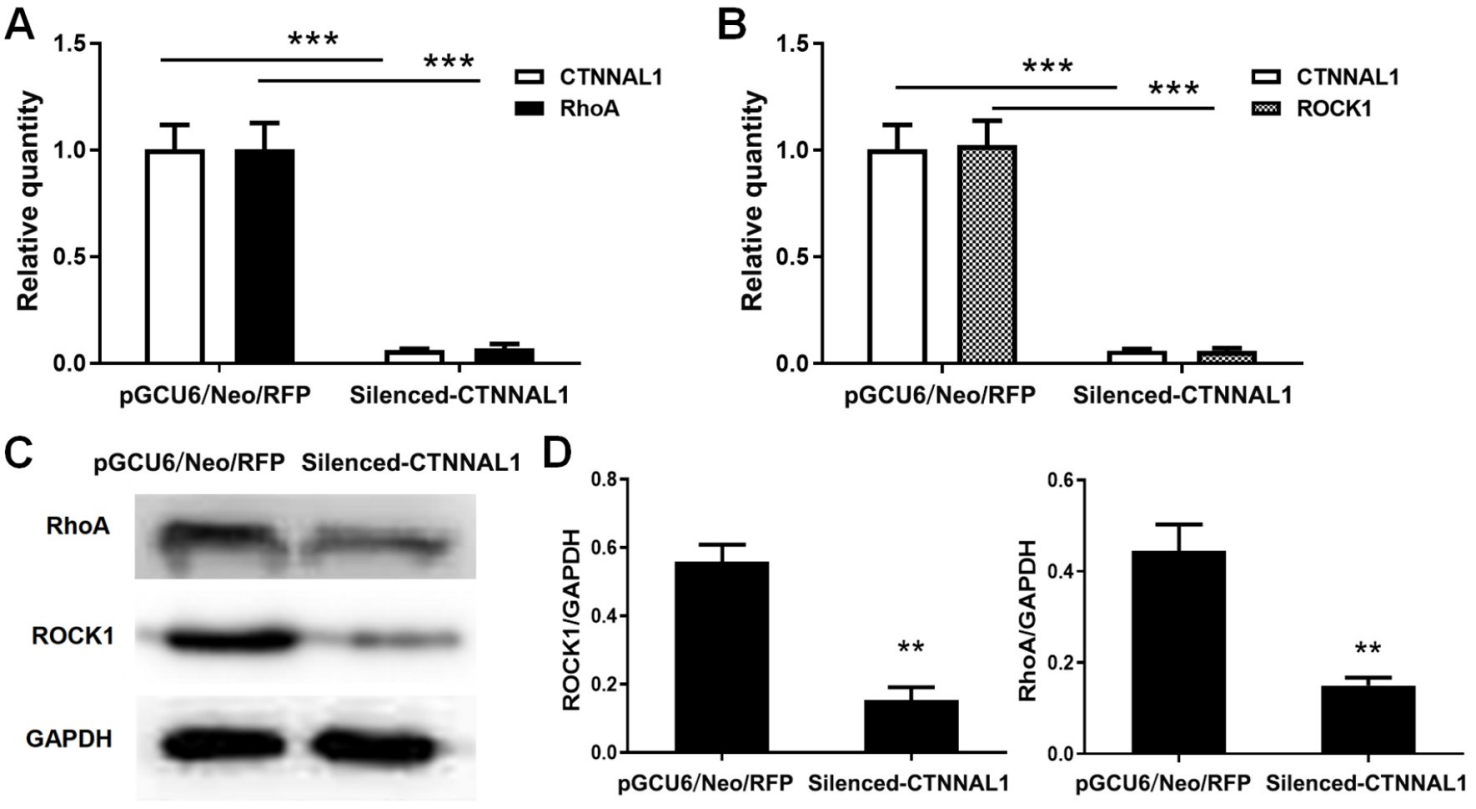
Figure 6 Effect of *CTNNAL1* knockdown on the expression of RhoA/ROCK1 (A) mRNA expressions of CTNNAL1, RhoA, and ROCK1 were examined by qRT-PCR. n=5. (B) Protein expressions of RhoA and ROCK1 were examined by western blot analysis. n=3. **P<0.01, ***P<0.001 vs pGCU6/Neo/RFP.

Rho GTPases play important roles in the organization of the actin cytoskeleton and are key elements for cell adhesion and morphology transformation [ 18– 20]. CTNNAL1 was identified as a scaffold protein for Lbc that participates in the Rho signaling pathway [13]. In the *CTNNAL1*-silenced group, the mRNA expression of RhoA was decreased by 92.97%, while that of ROCK1 was decreased by 94.35% ( Figure 6A,B). As expected, the protein levels of RhoA and ROCK1 were also significantly decreased ( Figure 6C,D).

**Figure 7:**
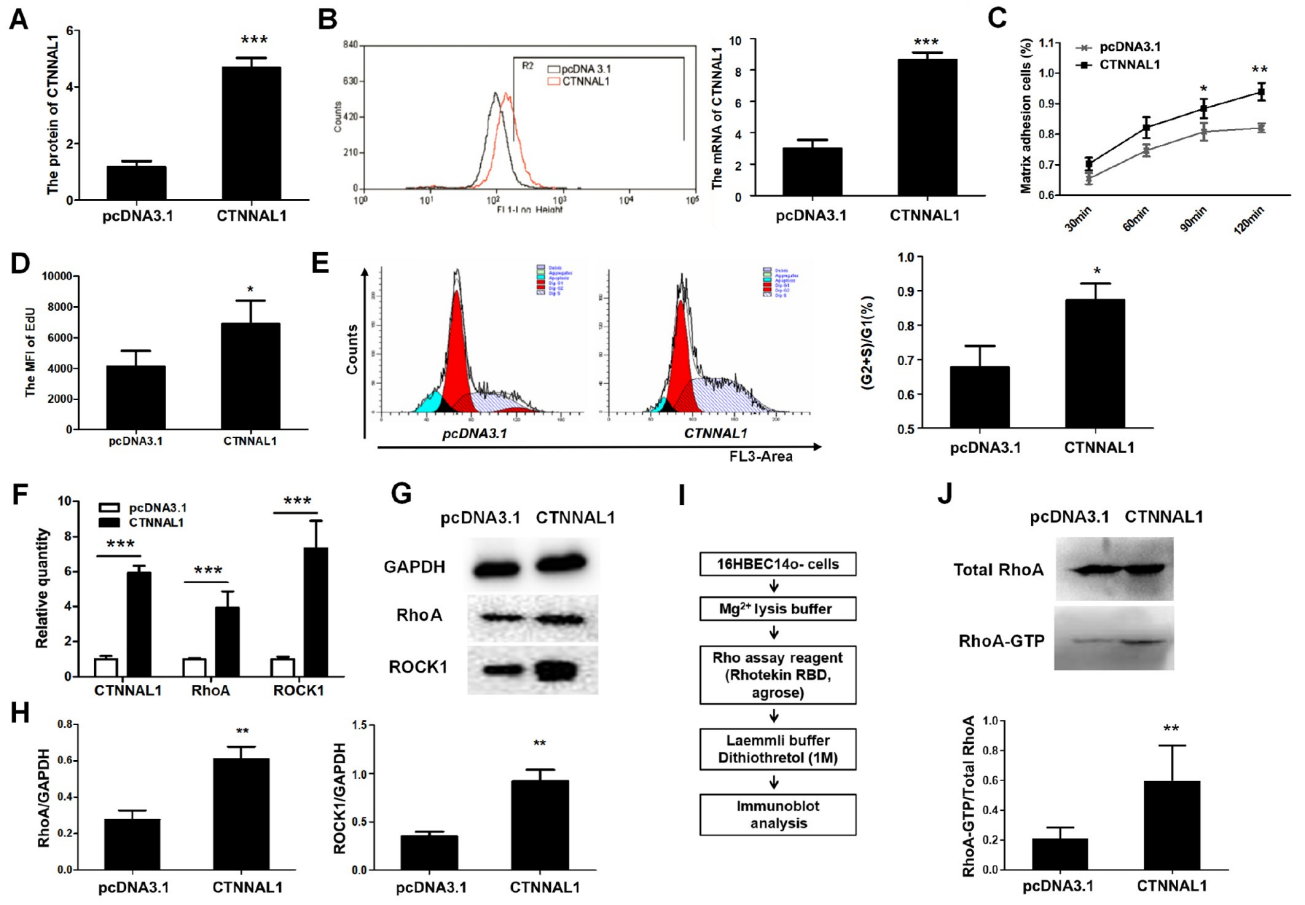
Figure 7 Effect of CTNNAL1 on the activity of RhoA To observe the effect of CTNNAL1 on the activity of RhoA, we constructed CTNNAL1-overexpressing (OE) cell lines. (A,B) mRNA and protein expressions of CTNNAL1 were evaluated by qRT-PCR and western blot analysis. (C) Percentage of epithelial CTNNAL1-OE cells adhering to rat tail collagen after 30 to 120 min. (D,E) Effect of CTNNAL1-OE on the proliferation of 16HBE14o- cells. (F‒H) The mRNA and protein expressions of RhoA and ROCK1 were assessed by qRT-PCR and western blot analysis, respectively. (I) In vitro pull-down of RhoA-GTP by the Rhotekin RBD and agrose. (J) RhoA activity was detected by western blot analysis and statistical analysis of RhoA activity. *P<0.05, **P<0.01, ***P<0.001 vs pcDNA3.1. n=3.

Because the expression of RhoA was significantly inhibited by silencing of *CTNNAL1*, little RhoA activity was detected in the *CTNNAL1*-silenced group. Therefore, we constructed CTNNAL1-overexpressing (OE) cell lines to observe the effect of CTNNAL1 on RhoA/ROCK1 signal transduction. The qRT-PCR ( Figure 7A) and FCM ( Figure 7B) results showed that the mRNA and protein expression levels of CTNNAL1 were significantly elevated by 493.5% and 399.51%, respectively, in the OE cells. As expected, the percentage of adherent cells with rat tail collagen in CTNNAL1-OE group continuously was increased over 120 min ( Figure 7C). In addition, increased expression of CTNNAL1 resulted in a notable increase in the MFI of EdU ( Figure 7D) and in the (G2+S)/G1 ( Figure 7E), indicating that CTNNAL1 has a proliferation-promoting effect on 16HBE14o- cells. These results demonstrated that the CTNNAL1-OE system was successful at detecting RhoA activity.

Next, we investigated the mRNA and protein expressions of RhoA and ROCK1 in OE cell lines by qRT-PCR and western blot analysis. As shown in Figure 7F‒H, the mRNA and protein expression levels of RhoA and ROCK1 were significantly increased by the overexpression of CTNNAL1. Then, we observed the function of RhoA in the OE cell line by pull-down assay and western blot analysis ( Figure 7I). As shown in Figure 7J, upregulated RhoA activity was observed in the CTNNAL1-OE group compared with that in the vector control group. The level of RhoA activity in the CTNNAL1-OE group was 3 times greater than that in the control group ( Figure 7J).

### ROCK inhibition abolished the CTNNAL1-induced effect on structural adhesion molecules

**Figure 8:**
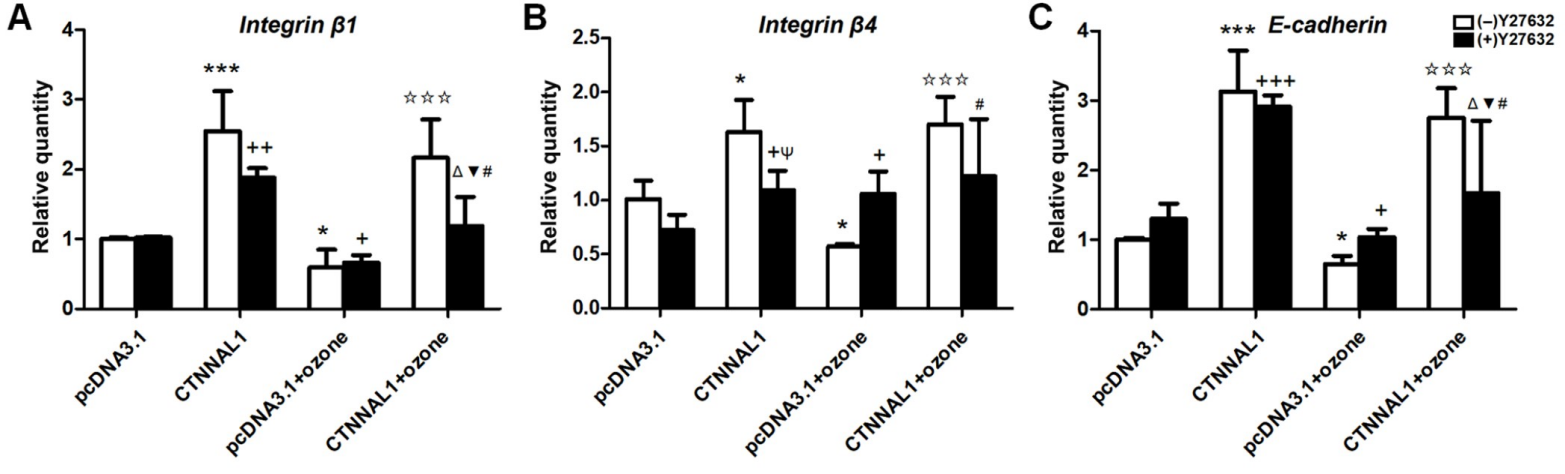
Figure 8 Pretreatment with Y27632 inhibited the CTNNAL1-induced increase in the expressions of adhesion molecules CTNNAL1-OE cells were pretreated with 20 μM Y27632 for 24 h. Then, the cells were collected for qRT-PCR analysis to evaluate the mRNA levels of the adhesion molecules integrin β1, integrin β4 and E-cadherin. (A) integrin β1. (B) integrin β4. (C) E-cadherin. *P<0.05, ***P<0.001 vs pcDNA3.1; ΨP<0.05 vs CTNNAL1; +P<0.05, ++P<0.01, +++P<0.001 vs pcDNA3.1+Y27632; ΔP<0.05 vs CTNNAL1+Y27632; ☆☆☆P<0.001 vs pcDNA3.1+ozone; #P<0.05 vs CTNNAL1+ozone; ▼P<0.05 vs pcDNA3.1+Y27632+ozone. n=3.

To examine whether the activation of RhoA/ROCK is involved in the CTNNAL1-induced effect on structural adhesion molecules, we pretreated the cells with Y27632 (a ROCK inhibitor). As shown in Figure 8, the mRNA levels of integrin β1, integrin β4, and E-cadherin were increased to 255.0%, 163.29%, and 313.0%, respectively, by the overexpression of CTNNAL1; however, these effects were markedly decreased by treatment with Y27632. Our data showed that Y27632 treatment did not affect the basal expressions of integrin β1, integrin β4, or E-cadherin in 16HBE14o- cells. These results indicated that Y27632 could inhibit the CTNNAL1-induced upregulation of structural adhesion molecules in 16HBE14o- cells. Interestingly, compared with those in pcDNA3.1 cells, the expressions of E-cadherin, integrin β1, and integrin β4 in pcDNA3.1+ ozone cells were significantly lower, but the ozone-induced decrease in the expressions of the three structural adhesion molecules was alleviated by the overexpression of CTNNAL1 ( Figure 8). However, Y27632 reversed the inhibitory effect of CTNNAL1 on the decrease in the expression of the three structural adhesion molecules induced by ozone, suggesting that CTNNAL1 upregulates the expressions of E-cadherin, integrin β1, and integrin β4 after ozone exposure through RhoA/ROCK1.

## Discussion

The airway epithelium forms a continuous, highly regulated physical barrier lining the airway lumen, which prevents invasion of inhaled environmental agents. The integrity of the airway epithelial barrier depends on the moderate expression of adhesion molecules in the airway epithelium [ 11, 12]. Adhesion molecules on BECs are involved in the stress response of the airway epithelium, and participate in the maintenance of epithelial structural integrity [1]. Abnormal expression of adhesion molecules in the airway epithelium may lead to disruption of epithelial integrity and promote proinflammatory activities in the epithelium. Ozone, a common inhaled air pollutant, is known to negatively impact respiratory health and contribute to exacerbations of asthma and increased hospitalizations [ 21, 22]. A decrease in the level of adhesion molecules (E-cadherin, integrin β1 and integrin β4), disruption of epithelial cell homologous adhesion, and rearrangement of the extracellular matrix are induced by ozone and are essential for airway hyperresponsiveness [ 23– 25]. In this study, we showed that silencing of *CTNNAL1* significantly weakened the structural adhesion ability of 16HBE14o- cells, as indicated by changes in the cell-matrix and cell-cell adhesion, suggesting that CTNNAL1 may be beneficial for maintaining airway integrity. After *CTNNAL1* silencing, the ozone-induced decreases in E-cadherin, integrin β1, and integrin β4 expressions in 16HBE14o- cells were greater than those in control cells, indicating the inhibitory effect of CTNNAL1 on the change in the expressions of structural molecules induced by ozone. Cell adhesion is inseparable from the regulation of actin cytoskeleton dynamics. Our study revealed that CTNNAL1 could affect the distribution of the F-actin cytoskeleton. In addition, CTNNAL1 promoted the mRNA and protein expressions of structural adhesion molecules, including E-cadherin, integrin β1, and integrin β4. E-cadherin is a major component of adherent junctions [26], and the adherence to junctions mediated by E-cadherin is closely associated with epithelial integrity [ 27, 28]. Additionally, integrin β1 and integrin β4, which are constitutively expressed on the surface of the basolateral airway epithelium, recognize the components of the basement membrane and mediate the anchorage of basolateral cells to the ECM [ 29, 30]. Together with E-cadherin, these proteins play multiple roles in the maintenance of cell architecture and structural integrity of the airway epithelium via the regulation of epithelial junctions, proliferation, and differentiation [31]. Like α-catenin, CTNNAL1 contains amphipathic helices in the C-terminal homology region, which are not only linked to growth signaling pathways, but also regulate cell proliferation [ 32, 33]. CTNNAL1 has been shown to affect cell migration, elevate cell resistance to apoptosis, and affect the *cyclin D1* promoter [ 34, 35]. Our data could partly explain the influence of CTNNAL1 on the structural adhesion of 16HBE14o- cells, which may be beneficial for maintaining airway integrity.

The actin cytoskeleton consists of a dynamic network of filaments that define and maintain cell morphology and regulate the dynamics of cell proliferation, adhesion, and motility [ 35– 37]. As a regulator of actin cytoskeleton dynamics, RhoA is the most well-studied member of the Rho GTPase family, and its activity regulates actin stress fiber assembly through the action of ROCK, which is downstream of RhoA and involved in inhibiting cell migration by enhancing adhesion to the ECM [ 38– 40]. CTNNAL1 is a component of the Rho signaling pathway and can interact with Lbc (a Rho-specific guanine nucleotide exchange factor) [ 13, 34]. Our results showed not only that the F-actin fiber level around the membrane in the *CTNNAL1*-silenced group was reduced and that its continuity was disrupted but also that the F-actin fiber level in the cell body was increased. We also found that CTNNAL1 significantly upregulated the expression of RhoA/ROCK1 and the activity of RhoA. These results indicate that CTNNAL1 may affect the reorganization of the actin cytoskeleton by regulating the RhoA/ROCK1 pathway. The RhoA/ROCK1 pathway participates in endothelial cell‒cell adhesion, endothelial permeability, and/or leukocyte transendothelial migration. Therefore, we further investigated adhesion molecule expression in 16HBE14o- cells pretreated with Y27632 (an inhibitor of ROCK1). Our results showed that the expressions of adhesion molecules induced by CTNNAL1 were inhibited by Y27632 treatment. The RhoA/ROCK1 pathway plays a role in cell-cell and cell-matrix adhesion by regulating the interaction between integrins, E-cadherin, and the actin cytoskeleton [ 40– 46]. Moreover, CTNNAL1 regulates the expression of cystic fibrosis transmembrane conductance regulator (CFTR) by ROCK1, thereby participating in mucus secretion of airway epithelial cells [47]. In our study, Y27632 itself did not affect the basal expression levels of adhesion molecules in the vector control group but inhibited the effect of CTNNAL1 on the expressions of adhesion molecules in 16HBE14o- cells induced by ozone. Here, our data demonstrated that the effects of CTNNAL1 on the expressions of adhesion molecules are dependent on the RhoA/ROCK1 pathway.

In summary, the finding of the present study demonstrates that CTNNAL1 plays a critical role in ozone-induced airway epithelial injury. Our results showed that CTNNAL1 promotes the structural integrity of the airway epithelium, increases the expressions of structural adhesion molecules in 16HBE14o- cells and is beneficial for maintaining the stability of the epithelial cytoskeleton, possibly through upregulating the expression and activity of RhoA/ROCK1. However, further *in vitro* and *in vivo* studies are needed to assess the modulatory effect of CTNNAL1 on ozone-induced cytoskeletal reorganization. Nevertheless, our findings provide new insights into the previously unidentified role of CTNNAL1 in maintaining airway structural integrity and highlight the potential of targeting CTNAAL1 in ozone-induced airway injury.
